# Copeptin concentration in cord blood in infants with early-onset sepsis, chorioamnionitis and perinatal asphyxia

**DOI:** 10.1186/1471-2431-11-38

**Published:** 2011-05-19

**Authors:** Luregn J Schlapbach, Stefanie Frey, Susanna Bigler, Chiem Manh-Nhi, Christoph Aebi, Mathias Nelle, Jean-Marc Nuoffer

**Affiliations:** 1 Neonatal and Pediatric Intensive Care Unit, Department of Pediatrics, University of Bern, 3010 Bern, Switzerland; 2 Institute for Infectious Diseases, University of Bern, 3010 Bern, Switzerland; 3 University Institute for Clinical Chemistry, University of Bern, 3010 Bern, Switzerland

**Keywords:** asphyxia, early-onset sepsis, copeptin, neonate, vasopressin

## Abstract

**Background:**

Vasopressin is one of the most important physiological stress and shock hormones. Copeptin, a stable vasopressin precursor, is a promising sepsis marker in adults. In contrast, its involvement in neonatal diseases remains unknown. The aim of this study was to establish copeptin concentrations in neonates of different stress states such as sepsis, chorioamnionitis and asphyxia.

**Methods:**

Copeptin cord blood concentration was determined using the BRAHMS kryptor assay. Neonates with early-onset sepsis (EOS, n = 30), chorioamnionitis (n = 33) and asphyxia (n = 25) were compared to a control group of preterm and term (n = 155) neonates.

**Results:**

Median copeptin concentration in cord blood was 36 pmol/l ranging from undetectable to 5498 pmol/l (IQR 7 - 419). Copeptin cord blood concentrations were non-normally distributed and increased with gestational age (p < 0.0001). Neonates born after vaginal compared to cesarean delivery had elevated copeptin levels (p < 0.0001). Copeptin correlated strongly with umbilical artery pH (Spearman's Rho -0.50, p < 0.0001), umbilical artery base excess (Rho -0.67, p < 0.0001) and with lactate at NICU admission (Rho 0.54, p < 0.0001). No difference was found when comparing copeptin cord blood concentrations between neonates with EOS and controls (multivariate p = 0.30). The highest copeptin concentrations were found in neonates with asphyxia (median 993 pmol/l). Receiver-operating-characteristic curve analysis showed that copeptin cord blood concentrations were strongly associated with asphyxia: the area under the curve resulted at 0.91 (95%-CI 0.87-0.96, p < 0.0001). A cut-off of 400 pmol/l had a sensitivity of 92% and a specifity of 82% for asphyxia as defined in this study.

**Conclusions:**

Copeptin concentrations were strongly related to factors associated with perinatal stress such as birth acidosis, asphyxia and vaginal delivery. In contrast, copeptin appears to be unsuitable for the diagnosis of EOS.

## Background

Arginine vasopressin (or antidiuretic hormone, ADH), is a nonapeptide acting as a main regulator in the homeostatis of the cardiovascular and renal system [1]. It is produced by the hypothalamus and secreted in the posterior lobe of the pituitary gland upon hemodynamic or osmotic stimuli. Vasopressin plays a crucial role in the endocrine stress response to a variety of diseases such as different shock states [2]. Exogenous vasopressin is a promising therapeutic agent in cardiac arrest and septic shock in adults [3]. A recent multicenter randomized controlled trial evaluated low-dose vasopressin in pediatric vasodilatory shock [4]. Although few authors have reported successful use of vasopressin in neonates with arterial hypotension [5-8], data on the role of vasopressin during the neonatal period are scarce.

Since vasopressin is highly instable with a short half-life of 4-20 minutes [1], reliable determination of vasopressin concentration is not used in clinical practice. Vasopressin is derived from a larger precursor peptide which contains also C-terminal pro-vasopressin, called copeptin [9]. Upon release of vasopressin, equal amounts of copeptin are secreted. Copeptin is relatively stable in serum and thereby reliably mirrors vasopressin levels [10,11]. Copeptin concentrations were strongly elevated in samples from adult patients with sepsis [9,12,13] and high copeptin levels were predictive of mortality [12,14]. Recent studies in adults have shown that copeptin is a valuable biomarker of infection in patients with community-acquired and ventilator-associated pneumonia [14].

Wellmann et al. have recently provided normal values for healthy term and near-term infants [15], but, to the best of our knowledge, copeptin has never been investigated in neonates with major diseases. We hypothesized that copeptin cord blood concentrations in neonates may be associated with different stress situations such as sepsis and perinatal asphyxia.

Neonatal infections account for over one million neonatal deaths worldwide every year [16]. Early-onset sepsis (EOS, presenting within 72 hours of age) occurs in approximately 0.6% of term and up to 1.5% of preterm infants and contributes significantly to neonatal mortality [17]. Therefore, early treatment of neonates with suspected infection is crucial to prevent life threatening complications. New infection markers may potentially improve guidance of therapeutic decisions [18].

Perinatal asphxia is, after prematurity and sepsis, the third main cause of neonatal death worldwide [19]. While therapeutic hypothermia has proven to improve survival and to reduce the rate of disability, mortality due to severe perinatal hypoxic-ischemic encephalopathy remains high even in developed countries [20].

This study therefore aimed to establish copeptin cord blood concentrations in neonates of different gestational ages and to assess the influence of sepsis, chorioamnionitis and asphyxia on copeptin concentrations.

## Methods

Infants born between November 2004 and November 2007 at the Department of Obstetrics, University of Bern, Switzerland, were eligible for this study if cord blood serum had been drawn and stored immediately after birth. Neonates with major congenital malformations were excluded. The study was approved by the institutional Ethical Board (Direktion Lehre und Forschung, Inselspital, University of Bern, 3010 Bern, Switzerland).

Patients and controls were searched using the institutional neonatal database. Perinatal characteristics and postnatal parameters were extracted from the institutional neonatal database. Arterial umbilical cord pH was obtained routinely. Blood gas analyses and hemoglobin concentrations were obtained routinely in neonates admitted to the neonatal intensive care unit (NICU) and were included in this study if they had been obtained within six hours after birth. Small for gestational age (SGA) was defined as infants with a birth weight below the 10^th ^percentile. Arterial hypotension was defined as mean arterial blood pressure which was below the gestational age limit at two consecutive measurements and that was treated with either a volume bolus administration, intravenous corticosteroids or catecholamines.

The following groups were defined: early-onset sepsis group, chorioamnionitis group, asphyxia group, and control group.

### Early onset sepsis group (EOS)

EOS cases were defined as neonates who presented with sepsis within the first 72 hours of life as defined by the following criteria [21]: i) at least two clinical signs of sepsis (temperature instability, irritability or apathy, feeding difficulties, poor capillary refill >2 seconds, apnea, tachycardia and/or tachypnea); ii) elevated C-reactive protein >20 mg/l, iii) decision of the attending physician to treat for at least 7 days with intravenous antibiotics and iv) recovery of bacterial pathogens in blood-culture. In infants with negative blood cultures but clinical diagnosis of EOS, all first three criteria mentioned above were required to be present.

### Chorioamnionitis group

Chorioamnionitis was defined as either clinically diagnosed chorioamnionitis (requiring presence of maternal fever, elevated maternal CRP, fetal tachycardia and prolonged rupture of membranes >24 h) or histologically diagnosed chorioamnionitis [22]. Only infants exposed to chorioamnionitis *without *evidence of neonatal infection as defined above were considered in order to avoid overlap with the EOS group.

### Asphyxia group

Asphyxia was defined according to our institutional guidelines as arterial cord blood pH below 7.1 plus 10-minute Apgar score below 6 and/or base excess >-12 mmol/l. This definition allowed to include both severe asphyxia [20], and milder forms of asphyxia. In addition, the asphyxia group required absence of clinically or histologically diagnosed chorioamnionitis and of confirmed EOS as defined above in order to avoid overlap with the EOS and the chorioamnionitis group. Hypoxic-ischemic encephalopathy (HIE) was graded according to Sarnat stage 0 (no HIE) to stage 3 (severe HIE) [23].

### Control group

Based on sample size calculations, a control group of at least 135 controls was required to detect a mean difference of 75 pmol/l between patients and controls with a power of 80% at a 95%-confidence interval. The assumptions were based on preliminary internal data and on published data from adults with sepsis [24]. The control group (n = 155) consisted of 75 premature neonates (24 0/7 to 36 6/7 weeks gestational age) without neonatal infection, chorioamnionitis or asphyxia (as defined above) and of 80 healthy term neonates (37 0/7 to 42 0/7 weeks gestational age) with no signs of any neonatal disease. Neonates with major congenital malformations were excluded. The control group infants were all born between January 2007 and November 2007.

### Copeptin measurements

Cord blood is routinely collected immediately after delivery of the child from the umbilical vein at the placental side of the cord, and is routinely stored at our institution to determine *Toxoplasma gondii *serology in neonates of mothers with unknown or negative serostatus. After centrifugation, cord blood serum was frozen in sterile tubes at -80°C. Copeptin cord blood concentrations were determined using the commercial BRAHMS copeptin kryptor assay according to the manufacturer's instructions (BRAHMS, Hennigsdort, Germany). The detection limit of the assay was 4.8 pmol/l.

### Statistical analysis

Copeptin concentrations were compared between patients and controls using Mann-Whitney U test and multivariate linear regression analysis. Multivariate analyses included gestational age, birth weight, SGA, mode of delivery and umbilical artery pH as covariates. Spearman's rank test and multivariate linear regression were used to analyze association between copeptin cord blood concentration with linear variables. Copeptin cord blood concentrations were logarithmized for regression analyses. Mann-Whitney U-test was used where appropriate. Receiver-operating-characteristic (ROC) curve analysis was used to assess specifity and sensitivity of copeptin for the diagnosis of EOS, chorioamnionitis and asphyxia. Two-sided tests were used throughout, and P-values below 0.05 were considered significant. SPSS 18.0 software was used.

## Results

### i) Patient characteristics

During the study period, 3896 neonates were born, 42 of whom fulfilled the EOS criteria. Cord blood serum was available in 30 (72%) neonates with EOS, these were thus included in the study. Their median gestational age was 31 weeks. In eight (27%) patients, blood cultures resulted positive. Maximum C-reactive protein during sepsis was at median 44 mg/l (range 22 - 261). Five infants (17%) required treatment with catecholamines due to septic shock and two (7%) infants died during sepsis. The chorioamnionitis group comprised 33 neonates without evidence of neonatal infection with a median gestational age of 30 weeks.

During the study period, 36 neonates were born with asphyxia. Of these, cord blood serum was available in 25 (69%). Their median gestational age was 37 weeks. Two (8%) had HIE Sarnat stage 3 and died, three (12%) Sarnat stage 2, and seven (28%) Sarnat stage 1, while 13 (52%) had no signs of HIE. Their median lactate concentration at NICU admission was 10.7 mmol/l (range 4 - 25).

Baseline characteristics of the patient groups and the control group are given in Table 1.

**Table 1:** Baseline characteristics according to study group

Study group	Early-onset sepsis (N = 30)	Chorioamnionitis (N = 33)	Asphyxia (N = 25)	Control (N = 155)
Gender (male)	12 (40%)	12 (36%)	10 (40%)	87 (56%)
Gestational age [weeks]	31.5 (29-34)	30 (28-34)	36.5 (33-39)	38 (32-40)
Birth weight [gram]	1665 (1161-2490)	1430 (998-1975)	2400 (1573-3213)	2800 (1590-3420)
Prenatal steroids	23 (77%)	27 (82%)	8 (32%)	64 (41%)
Cesarean section	18 (60%)	18 (55%)	17 (68%)	73 (47%)
PROM >24 h	7 (23%)	10 (30%)	2 (8%)	16 (10%)
SGA	3 (10%)	4 (12%)	7 (28%)	11 (7%)
APGAR 1 min	5 (3-7)	6 (4-8)	3 (2-6)	8 (6-8)
APGAR 5 min	8 (6-9)	8 (7-9)	7 (5-8)	9 (8-9)
APGAR 10 min	9 (7-9)	9 (8-9)	8 (7-9)	9 (9-9)
Cord blood arterial pH	7.30 (7.24-7.33)	7.29 (7.24-7.33)	7.03 (7.00-7.07)	7.29 (7.25-7.32)
PDA	6 (20%)	5 (15%)	0 (0%)	11 (7%)
Arterial hypotension	12 (40%)	11 (33%)	11 (44%)	32 (21%)
Mechanical ventilation	16 (53%)	13 (39%)	8 (32%)	13 (8%)

Median (interquartile range) or number (percentage) are shown.

PDA, persistent ductus arteriosus; PROM, prolonged rupture of membranes; SGA, small for gestational age (< 10^th ^percentile)

### ii) Copeptin cord blood concentrations in the whole cohort

**Figure 1:**
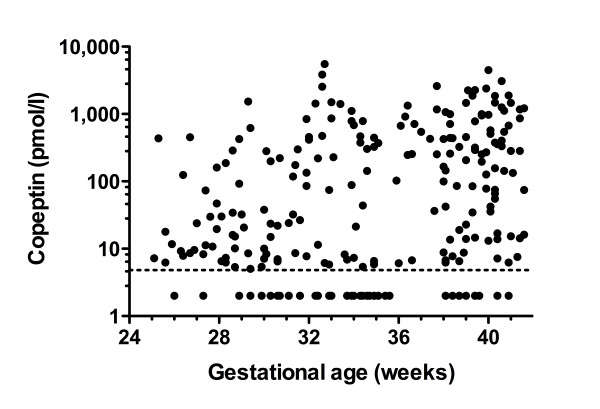
**Copeptin and gestational age**. Copeptin cord blood concentrations are shown according to gestational age. The dotted line indicates the detection limit (4.8 pmol/l).

**Figure 2:**
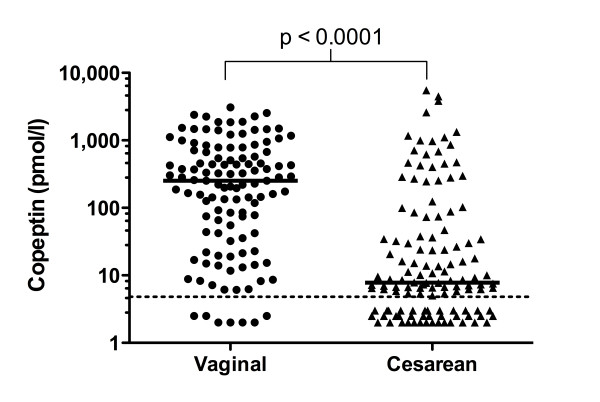
**Copeptin concentration is increased after vaginal delivery**. Copeptin cord blood concentrations according to the delivery mode are shown. The medians and the p-value of Mann-Whitney U test are shown. The dotted line indicates the detection limit (4.8 pmol/l).

**Figure 3:**
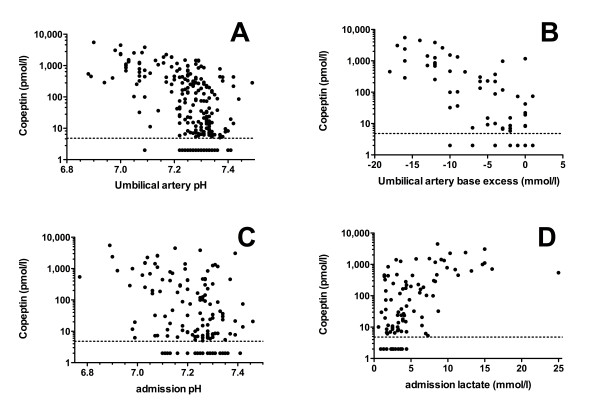
**Copeptin concentration correlates with perinatal acidosis**. Copeptin cord blood concentrations are plotted against umbilical artery pH (**A**, n = 236), umbilical artery base excess (**B**, n = 60), pH at admission to the NICU (**C**, n = 138) and lactate concentration at admission to the NICU (**D**, n = 100). The dotted line indicates the detection limit (4.8 pmol/l).

When analyzing copeptin cord blood concentrations in the whole study population (n = 243), median concentration was 36 pmol/l ranging from undetectable to 5498 pmol/l (IQR 7 - 419). Copeptin concentration correlated significantly with gestational age (Spearman's Rho 0.30, p < 0.0001, Figure 1), and birth weight (Rho 0.29, p < 0.0001), but did not differ between boys and girls (Mann-Whitney Z -10.98, p = 0.33). Infants after vaginal delivery compared to cesarean delivery had significantly higher copeptin levels (Mann-Whitney Z -7.32, p < 0.0001, Figure 2), even when adjusting for gestational age (p < 0.001). Copeptin cord blood concentration showed a strong negative correlation with umbilical artery pH (Rho -0.50, p < 0.0001) and umbilical artery base excess (Rho -0.67, p < 0.0001), see Figure 3. Similarly, copeptin concentration correlated strongly with pH (Rho -0.34, p < 0.0001) and with lactate at NICU admission (Rho 0.54, p < 0.0001), see Figure 3. The correlations between copeptin and pH, base excess and lactate remained significant in subgroup analyses on very preterm, late preterm and term neonates (< 32, 32-36 6/7, ≥ 37 weeks gestational age, p < 0.05, details not shown).

No association was found between copeptin cord blood concentrations and arterial hypotension requiring treatment with volume and/or vasopressors, or with hemoglobin concentration and hematocrit at NICU admission (p >0.05).

### iii) Copeptin cord blood concentrations in neonates with EOS and with chorioamnionitis

**Figure 4:**
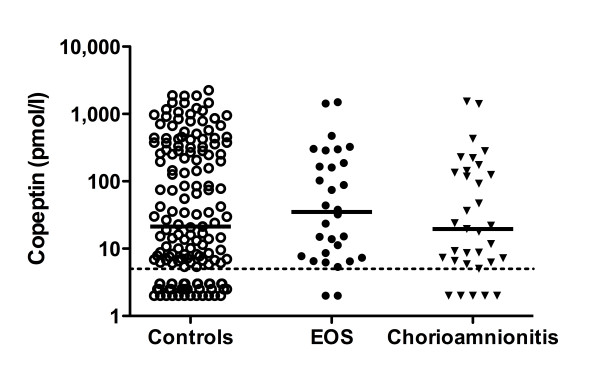
**Copeptin concentration and early-onset sepsis**. Copeptin cord blood concentrations in neonates with early-onset sepsis (EOS) and chorioamnnionitis compared with controls. The medians are shown. The dotted line indicates the detection limit (4.8 pmol/l).

Median copeptin concentrations were 35 pmol/l (IQR 8 - 212) in the EOS versus 20 pmol/l (IQR 6 - 139) in the chorioamnionitis group versus 21 pmol/l (IQR 5 - 324) in controls, see Figure 4. Although median copeptin concentrations were higher in EOS infants compared to controls, this was not statistically significantly (Mann-Whitney Z -0.58, p = 0.56). This was confirmed by multivariate linear regression analysis adjusted for gestational age, birth weight, SGA, delivery mode and umbilical artery pH (beta coefficient 0.16, 95%-CI -0.14 - 0.45, p = 0.30), Copeptin concentrations did not significantly differ between EOS infants with septic shock or with positive blood cultures compared to the rest of EOS infants (p >0.05). ROC curve analysis confirmed that the performance of copeptin to distinguish EOS from controls was poor (area under the curve, 0.53, 95%-CI 0.44 - 0.63, p = 0.57). No difference was found when comparing copeptin levels between the chorioamnionitis group and controls or between the chorioamnionitis and the EOS group (p >0.1). Copoeptin was not associated with CRP, leukocyte count or left shift (immature by total neutrophil ratio, p >0.05, details not shown).

### iv) Copeptin cord blood concentrations in neonates with asphyxia

**Figure 5:**
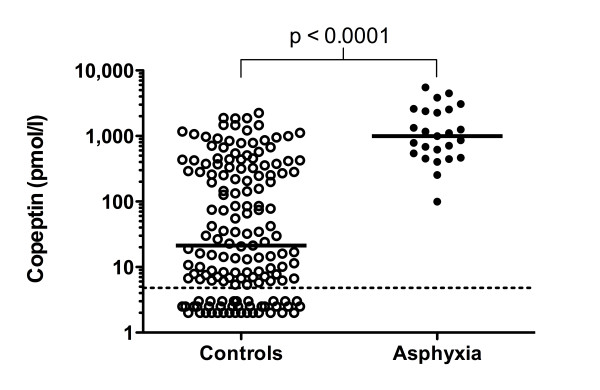
**Copeptin concentration in infants with asphyxia**. Copeptin cord blood concentrations in neonates with asphyxia compared with controls. The medians and the p-value of Man-Whitney U test are shown. The dotted line indicates the detection limit (4.8 pmol/l).

Copeptin cord blood concentrations were significantly higher in the 25 neonates with asphyxia (median 993 pmol/l, IQR 505 - 2466, range 100 - 5498) compared to controls (Mann-Whitney Z -6.49, p < 0.0001), see Figure 5. This was confirmed by multivariate analysis (beta coefficient 1.09, 95%-CI 0.41 - 1.76, p = 0.002). Notably, the eight highest copeptin values measured, all above 2000 pmol/l, occurred in neonates with asphyxia. None of these eight neonates with very high copeptin levels had more than Sarnat stage one HIE, and all survived. Copeptin concentration was not significantly correlated with Sarnat score (Rho -0.31, p = 0.133).

**Figure 6:**
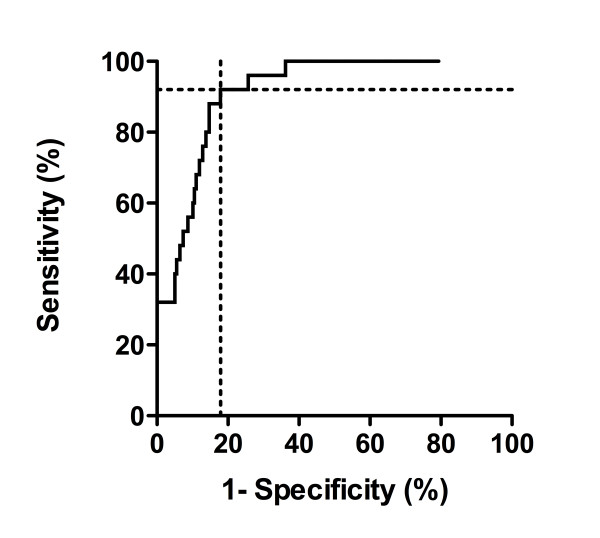
**Receiver-operating-characteristic curve of copeptin concentrations in relation to asphyxia**. Receiver-operating-characteristic curve is shown for copeptin cord blood concentrations in relation to asphyxia. The dotted lines indicate the optimal discriminative cut-off of 400 pmol/l, resulting in a sensitivity of 92% and a specifity of 82%.

ROC curve analysis showed that copeptin concentrations discriminated with high accuracy between asphyxia, as defined in this study, and controls: the area under the curve resulted at 0.91 (95%-CI 0.87 - 0.96, p < 0.0001, see Figure 6). A cut-off of 400 pmol/l had a sensitivity of 92% and a specifity of 82%.

## Discussion

Our findings indicate that copeptin cord blood concentrations reflect perinatal stress with the highest values found in neonates with asphyxia. To the best of our knowledge, this is the first study to investigate copeptin in newborns with EOS and asphyxia.

Copeptin cord blood concentration was strongly and inversely correlated with umbilical artery pH, umbilical artery base excess, and with pH and lactate at admission to the NICU. Perinatal acidosis results from diminished fetal blood and oxygen supply due to maternal, placental or cord complications leading to lactic acidosis. Vasopressin is released by the hypothalamus-hypopituitary upon sensing of increased plasma osmolality, decreased arterial pressure, and reductions in cardiac volume [1]. Our data indicate that the vasopressin system in the neonate is strongly activated upon perinatal stress. Importantly, the strength of these correlations was comparable between very preterm, late preterm and term infants, suggesting that the vasopressin response is already present at an early gestational age.

The highest copeptin cord blood concentrations were found in neonates with perinatal asphyxia. This finding was confirmed by multivariate analysis adjusted for gestational age, birth weight, mode of delivery and umbilical artery pH. Most values in this group, a third of them exceeding 2000 pmol/l, were much higher that concentrations that have been reported in adult studies on patients with septic shock, multiple trauma or myocardial infarction [9,25,26]. Copeptin cord blood concentrations above 400 pmol/l had a high sensitivity and specifity for asphyxia. Perinatal asphyxia can be considered as an extreme of a stress situation. In a study on the stress response to hypoxia in neonatal piglets, maintenance of cardiovascular function and a higher serum cortisol concentration were associated with a better neurological outcome [27]. Further studies are needed to determine whether copeptin is related to asphyxia severity and whether copeptin may improve outcome prognostication after asphyxia.

Neonates born by vaginal delivery had significantly elevated copeptin cord blood concentrations compared to those born by cesarean section, even after adjustment for gestational age. Wellmann et al. have recently determined copeptin in 177 neonates and found higher levels after vaginal delivery as well [15]. These findings are in line with earlier reports of elevations in the stress hormone cortisol after vaginal delivery [28]. Spontaneous labour physiologically induces important changes in the fetal homeostatic system which serve to prime the fetus for postnatal adaptation.

Copeptin has been shown to be a valuable infection marker in adults with sepsis and community-acquired pneumonia [9,12-14]. In contrast, in our study, copeptin concentrations in cord blood were not significantly elevated in EOS infants compared to controls. The specifity and sensitivity of copeptin in the diagnosis of EOS was poor. Similarly, no difference was found between neonates born to mothers with chorioamnionitis and controls. Given the strong influence of perinatal stress on copeptin cord blood concentrations, and considering the very large interindividual variations observed in this study, our data indicate that determining copeptin concentrations is not suitable to diagnose EOS. Potentially, the diagnostic accuracy of copeptin may be improved in late-onset infections, since the effect of perinatal stress on copeptin disappears over the first days of life [15].

Several limitations of this study need to be mentioned. Firstly, only neonates where cord blood was available were included. A selection bias is, however, unlikely, since cord blood was routinely collected during the study period in neonates of mothers with unknown or negative *Toxoplasma gondii *serostatus, a condition which is unlikely to affect copeptin cord blood concentrations. Secondly, the relatively small sample sizes limits statistical power. Therefore, confirmation by future prospective cohorts is needed.

We believe that the present study has several strengths. In contrast to the study by Wellmann et al. [15] which included only healthy term and near term infants, we included neonates with a wide range of gestational ages. The inclusion of clearly defined and not overlapping groups of infants with EOS, chorioamnionitis and asphyxia allowed to study the influence of these diseases on copeptin concentration. Multivariate analyses were adjusted for the main confounders gestational age, birth weight, delivery mode and umbilical artery pH.

## Conclusions

We report that copeptin concentrations in cord blood are strongly correlated to perinatal stress with the highest values found in neonates with perinatal asphyxia. Future studies should prospectively determine copeptin concentrations in combination with novel markers of neonatal brain damage, such as neuron-specific enolase or S-100B [29-31], in order to investigate whether copeptin concentrations are of prognostic value during asphyxia.

## Abbreviations

CRP: C-reactive protein; EOS: early-onset sepsis; HIE: hypoxic-ischemic encephalopathy; ROC: receiver-operating characteristic

## Competing interests

The authors declare that they have no competing interests.

## Authors' contributions

LS had primary responsibility for the study design, data acquisition, analysis and writing the manuscript. SF performed clinical data collection, statistical analyses and helped to draft the manuscript. CA and MN were involved in study design, data acquisition and writing of the manuscript. SB, CM and JMN carried out processing of umbilical cord sera, performed the laboratory analyses and helped to draft the manuscript. All authors have read and approved the final manuscript.

## Pre-publication history

The pre-publication history for this paper can be accessed here:

http://www.biomedcentral.com/1471-2431/11/38/prepub
